# SHMT2 Suppression Disrupts Redox Homeostasis and Induces Ferroptosis Through the GSH–GPX4 Axis in Head and Neck Cancer

**DOI:** 10.3390/antiox15101300

**Published:** 2026-10-08

**Authors:** Shan Shen, Mi Ae Lim, Sicong Zheng, Shuyu Piao, Doh Hoon Kim, Shengzhe Cui, Young Il Kim, Jae Won Chang, Seung-Nam Jung, Bon Seok Koo

**Affiliations:** 1 Department of Otolaryngology-Head and Neck Surgery, College of Medicine, Chungnam National University, Daejeon 35015, Republic of Korea; shenshan8656@163.com (S.S.);; 2 Department of Medical Science, College of Medicine, Chungnam National University, Daejeon 35015, Republic of Korea; 3 Department of Radiation Oncology, Chungnam National University, Sejong Hospital, Sejong 30099, Republic of Korea

**Keywords:** *SHMT2*, GSH–GPX4 axis, ferroptosis, lipid peroxidation, head and neck cancer

## Abstract

Head and neck squamous cell carcinoma (HNSCC) remains one of the most prevalent and aggressive malignancies worldwide. Serine hydroxymethyltransferase 2 (SHMT2), a key enzyme in one-carbon metabolism, is essential for tumor cell proliferation and redox homeostasis. Whether *SHMT2* regulates ferroptosis, an iron-dependent form of regulated cell death, in HNSCC remains unknown. Here, we demonstrate that SHMT2 suppression induces ferroptotic cell death in HNSCC cells, as evidenced by increased lipid peroxidation and elevated prostaglandin-endoperoxide synthase 2 (PTGS2, also known as COX-2) expression. Mechanistically, SHMT2 suppression decreased the intracellular GSH/GSSG ratio, while exogenous GSH partially restored cell viability and attenuated lipid peroxidation. Moreover, SHMT2 suppression reduced GPX4 protein levels, whereas *SHMT2* overexpression increased GPX4 abundance. Conversely, *GPX4* overexpression had little effect on SHMT2, placing SHMT2 upstream of GPX4. Functionally, *GPX4* overexpression partially restored cell viability and reduced lipid peroxidation in *SHMT2*-knockdown cells, further supporting a functional role for GPX4 downstream of SHMT2. Both genetic and pharmacological inhibition of SHMT2 enhanced erastin-induced ferroptosis in vitro, while SHMT2 knockdown potentiated the antitumor activity of erastin in female BALB/c nude mouse xenografts. Collectively, our study identifies a novel *SHMT2*-regulated GSH–GPX4 axis that mediates ferroptosis in HNSCC and highlights SHMT2 suppression as a potential therapeutic strategy for head and neck cancer.

## 1. Introduction

Head and neck squamous cell carcinoma (HNSCC) is the seventh most common malignancy worldwide, accounting for more than 660,000 new cases and approximately 325,000 deaths annually [1,2]. Owing to the absence of obvious clinical symptoms at early stages, the majority of patients are diagnosed at an advanced stage, and the five-year survival rate remains below 65% [3]. Although substantial advances have been achieved in radiotherapy and surgical techniques, together with the application of chemotherapy and monoclonal antibody therapies, more than half of patients still experience disease recurrence following treatment [4,5]. Consequently, molecular characterization has become increasingly important for prognostic stratification and the development of personalized therapeutic strategies in HNSCC [6]. Identifying key molecular biomarkers involved in HNSCC initiation and progression is therefore of great importance for improving diagnosis and prognostic assessment.

Serine hydroxymethyltransferase 2 (*SHMT2*) is a key mitochondrial enzyme involved in one-carbon metabolism that catalyzes the conversion of serine to glycine while generating tetrahydrofolate-bound one-carbon units, thereby supporting nucleotide biosynthesis and promoting tumor cell proliferation [7]. Previous studies have demonstrated that SHMT2 is highly expressed in a wide range of malignancies, including B-cell lymphoma, thyroid cancer, colorectal cancer, and HNSCC, where it exerts oncogenic functions in tumor development and progression [8,9,10]. Our recent work further revealed that SHMT2 facilitates HNSCC progression by promoting cancer stemness [11]. With the expanding understanding of SHMT2 biology, it has emerged as a promising therapeutic target. Although emerging evidence indicates that SHMT2 regulates oxidative stress–induced cell death modes such as apoptosis [12,13], its precise role in regulating ferroptosis—an iron-dependent form of lipid peroxidative cell death—remains poorly understood in head and neck cancer.

Ferroptosis is an iron-dependent form of programmed cell death characterized by excessive lipid peroxidation and oxidative damage to cellular membranes, and is mechanistically and morphologically distinct from apoptosis and necrosis [14]. As an important tumor-suppressive mechanism, ferroptosis has been shown to play a critical role in tumor initiation and progression [15]. In recent years, ferroptosis has attracted increasing attention as a promising strategy to overcome therapeutic resistance, since therapy-resistant and metastatic tumor cells exhibit heightened sensitivity to ferroptosis [16].

Cells have evolved multiple defense systems to counteract lipid peroxidation–induced ferroptotic toxicity, including the xCT–GSH–GPX4 axis, the ferroptosis suppressor protein 1 (FSP1)–CoQ10–NAD(P)H axis, the GTP cyclohydrolase 1 (GCH1)/BH4 axis, and the dihydroorotate dehydrogenase (DHODH)/CoQH2 axis [17]. Among these, the GPX4-mediated xCT–GSH–GPX4 axis represents the principal cellular defense mechanism against ferroptosis. GPX4 acts as a lipid peroxide reductase that reduces toxic lipid hydroperoxides in cellular membranes to non-toxic lipid alcohols, thereby preventing the execution of ferroptosis [18]. The classical ferroptosis inducer erastin potently triggers oxidative cell death by inhibiting the cystine/glutamate antiporter xCT, resulting in glutathione (GSH) depletion and subsequent functional inactivation of GPX4 [19,20]. Notably, glutathione (GSH) is synthesized from glutamate, cysteine, and glycine, and SHMT2 serves as a key enzyme responsible for intracellular glycine production.

Emerging evidence has linked mitochondrial SHMT2 to cellular redox maintenance, with recent studies showing that SHMT2 suppresses ferroptosis in non-malignant contexts such as hematopoietic stem cells [21]. However, whether SHMT2 regulates ferroptotic susceptibility in head and neck cancer remains unexplored.

Accordingly, we hypothesized that inhibition of SHMT2 may induce ferroptosis by modulating the GSH–GPX4 axis [22]. In this study, we identify SHMT2 as a critical regulator of ferroptosis in HNSCC via the GSH–GPX4 axis. We further demonstrate that otherwise ineffective doses of erastin robustly induce ferroptosis upon SHMT2 knockdown, establishing SHMT2 as a key determinant of ferroptosis susceptibility. Consistently, SHMT2 suppression enhances erastin cytotoxicity in HNSCC cell lines and enhances the antitumor effect of erastin in a nude mouse xenograft model.

## 2. Materials and Methods

### 2.1. Cell Lines and Culture Conditions

The human head and neck squamous cell carcinoma (HNSCC) cell lines SNU1041 and MSK-QLL1 were obtained from the Korea Cell Line Bank (KCLB, Seoul, Republic of Korea). SNU1041 is a human hypopharyngeal squamous cell carcinoma cell line originally established at Seoul National University, whereas MSK-QLL1 is a human HNSCC cell line originating from Memorial Sloan Kettering Cancer Center. These cell lines were selected as representative HNSCC models because they originate from distinct backgrounds, exhibit endogenous SHMT2 expression, and showed reliable growth and experimental performance in our preliminary experiments. The use of two independent HNSCC cell lines allowed us to assess the reproducibility of the observed effects across different cellular models. SNU1041 cells were maintained in RPMI-1640 (Welgene, Gyeongsan, Republic of Korea), while MSK-QLL1 cells were propagated in DMEM/F12 (Welgene). Both media were supplemented with 10% fetal bovine serum (FBS; Gibco, Grand Island, NY, USA) and 1% penicillin–streptomycin (Gibco). Cultures were sustained in a humidified incubator at 37 °C under 5% CO_2_. To ensure experimental reproducibility and prevent phenotypic drift, all assays were conducted using cells within passages 5 to 10.

### 2.2. Inhibitors and Chemical Reagents

The selective *SHMT1/2* inhibitor, SHIN2 (Cat. No. AOB11286; 98% purity), was acquired from Aobious (Gloucester, MA, USA). The ferroptosis-inducing agent, erastin (Cat. No. HY-15763; 99.62% purity), was provided by Med Chem Express (MCE, Monmouth Junction, NJ, USA). Stock solutions were prepared by dissolving compounds in anhydrous DMSO and stored at −20 °C. For all functional assays, the final concentration of DMSO was restricted to <0.1% (*v*/*v*) to preclude any potential vehicle-induced cytotoxicity.

### 2.3. Bioinformatics Analysis

The Cancer Genome Atlas head and neck squamous cell carcinoma (TCGA-HNSC) dataset was obtained through the Genomic Data Commons (GDC) portal (https://portal.gdc.cancer.gov/ (accessed on 15 December 2025)). The dataset included RNA-seq data from 520 primary tumor tissues and 44 adjacent normal tissues. Gene expression data were obtained as raw read count matrices and transcripts per million (TPM) values for downstream analyses. In addition, gene expression differences between tumor and normal tissues were further validated using the UALCAN online platform (http://ualcan.path.uab.edu (accessed on 15 December 2025)), which provides TCGA-based expression analysis and visualization.

Differential gene expression analysis between tumor tissues and normal tissues in the TCGA-HNSC cohort was performed using the “DESeq2” R package (version 1.46.0) in R (version 4.4.3). Volcano plots were generated using the “ggpubr” R package (version 0.6.0). To further investigate the association between SHMT2 expression and transcriptional alterations in TCGA-HNSC tumors, tumor samples were stratified into high- and low-SHMT2 expression groups based on SHMT2 expression levels derived from the TPM-normalized gene expression matrix. Differentially expressed genes between the two groups were identified using the same DESeq2 pipeline, and the resulting gene lists were used for subsequent analyses.

The 18 regulated cell-death pathways evaluated in this study were selected with reference to published cell-death classifications and literature-curated gene sets [23,24,25,26]. Gene set enrichment analysis (GSEA) was performed using the “fgsea” package in R (version 4.4.3; fgsea version 1.32.4), and enrichment results were visualized using “GseaVis” (version 0.0.5).

### 2.4. Human Tissue Samples

Human tumor specimens were collected from nine patients with head and neck squamous cell carcinoma (HNSCC) undergoing surgical resection at Chungnam National University Hospital (CNUH). Paired tumor tissues and adjacent normal tissues were obtained from each patient. All samples were collected after written informed consent in accordance with institutional guidelines. The study protocol was approved by the Institutional Review Board of Chungnam National University Hospital (CNUH IRB No. 2025-05-057; approved on 28 May 2025).

### 2.5. RNA Isolation and Quantitative Real-Time PCR (qRT-PCR)

Total RNA was isolated from SNU1041 and MSK-QLL1 cells using TRIzol™ Reagent (Cat. No. 15596018; Invitrogen, Carlsbad, CA, USA) according to the manufacturer’s instructions. For cDNA synthesis, 2 μg of total RNA was reverse- transcribed using TOPscript™ RT DryMIX (Enzynomics, Daejeon, Republic of Korea) in a final volume of 20 μL. Quantitative real-time PCR (qRT-PCR) was performed using SYBR Green qPCR Master Mix (Thermo Fisher Scientific, Waltham, MA, USA) on a standard real-time PCR system. Relative mRNA expression was calculated using the 2^−ΔCt^ or 2^−ΔΔCt^ method, as appropriate, with GAPDH used as the endogenous internal control. The specific primer sequences are listed in Supplementary Table S1.

### 2.6. Cell Migration Assay

Cell migration was assessed using a Transwell migration assay. SHMT2 knockdown SNU1041 and MSK-QLL1 cells, along with their corresponding control cells, were cultured in complete medium until reaching approximately 70–80% confluence. Cells were then serum-starved and seeded at a density of 3 × 10^3^ cells per insert in 200 μL of serum-free medium into the upper chambers of Transwell inserts (Corning^®^ Costar^®^, Cat. No. 3492; Corning Incorporated, Corning, NY, USA). The lower chambers were filled with 500 μL of medium containing 10% fetal bovine serum (FBS) to serve as a chemoattractant. Cells were allowed to migrate for 48 h at 37 °C in a humidified atmosphere containing 5% CO_2_. After incubation, non-migrated cells remaining on the upper surface of the membrane were gently removed using a cotton swab. Migrated cells attached to the lower surface were fixed with 4% paraformaldehyde for 15 min and stained with 0.1% crystal violet for 10 min. The membranes were subsequently rinsed with phosphate-buffered saline (PBS) and air-dried. Images were acquired using an inverted microscope (Nikon Eclipse Ti; Nikon Corporation, Tokyo, Japan) equipped with a 10× Plan Achromat objective. Migrated cells were quantified by counting at least three randomly selected fields per insert. Each experiment was performed independently in triplicate.

### 2.7. Western Blot Analysis

Total protein was extracted from cells and tumor tissues using RIPA lysis buffer (LPS Solution, Daejeon, Republic of Korea) supplemented with protease and phosphatase inhibitors (Thermo Fisher Scientific, Waltham, MA, USA). Protein concentrations were determined using a BCA assay (Thermo Fisher Scientific). Equal amounts of protein were separated by SDS–PAGE and transferred onto PVDF membranes. Membranes were incubated overnight at 4 °C with primary antibodies against SHMT2 (1:1000, Cell Signaling Technology [CST], Danvers, MA, USA), GPX4 (1:1000, CST, Danvers, MA, USA), FSP1 (1:1000, CST), DHODH (1:1000, CST), xCT/SLC7A11 (1:1000, CST), GCH1 (1:500, Abcam, Cambridge, UK), 4-hydroxynonenal (4-HNE; 1:1000, Abcam; Cat. No. ab46545), and *β*-actin (1:1000, CST). After incubation with HRP-conjugated secondary antibodies (Santa Cruz Biotechnology, Dallas, TX, USA), immunoreactive bands were visualized using FemtoMax™ enhanced chemiluminescence substrate (LPS Solution, Daejeon, Republic of Korea). Protein levels were normalized to *β*-actin and quantified by densitometric analysis using ImageJ software (Fiji 2.14.0, ImageJ 1.54i; National Institutes of Health, Bethesda, MD, USA). All experiments were performed in triplicate.

### 2.8. Lentiviral shRNA-Mediated Knockdown of SHMT2

To investigate the functional role of SHMT2, shRNA-mediated knockdown models were established in SNU1041 and MSK-QLL1 cells. Cells were infected with lentiviral particles encoding SHMT2-specific shRNAs (OriGene Technologies, Rockville, MD, USA; Cat. No. TL309453V). A non-targeting scrambled shRNA lentiviral particle was used as a negative control (OriGene Technologies; Cat. No. TR30021V). Lentiviral infection was performed at a multiplicity of infection (MOI) of 20 for 24 h in the presence of 8 μg/mL polybrene. After infection, the medium was replaced with fresh complete medium, and cells were selected with puromycin (2 μg/mL) for 3–5 days to establish stable knockdown cell lines. Knockdown efficiency was confirmed by qRT–PCR and Western blot analysis. The sequences of shRNAs targeting SHMT2 are listed in Supplementary Table S2.

### 2.9. Cell Transfection with Overexpression Vectors

To investigate the effects of SHMT2 and GPX4 overexpression, human *SHMT2* (RC204239) and *GPX4* (RG208065) expression plasmids were purchased from OriGene Technologies (Rockville, MD, USA). Cells were seeded in plates and transfected with the indicated expression plasmids or empty vector using jetPEI transfection reagent (Polyplus-transfection) according to the manufacturer’s instructions. Briefly, plasmid DNA was diluted in serum-free medium and mixed with jetPEI reagent to allow complex formation at room temperature. The DNA–jetPEI complexes were then added directly to the cells without medium replacement. Cells were incubated at 37 °C in a humidified atmosphere containing 5% CO_2_.

### 2.10. Colony Formation Assay

SNU1041 and MSK-QLL1 cells expressing shNC, shSHMT2 #1, or shSHMT2 #2 were seeded in six-well plates at a density of 1500 cells per well and cultured for 10 days. Colonies were fixed with 4% paraformaldehyde for 15 min and stained with 1% crystal violet for 20 min. After washing and air-drying, colonies were quantified using ImageJ. Each experiment was performed independently three times.

### 2.11. Direct Cell Counting Assay

SNU1041 and MSK-QLL1 cells expressing shNC, shSHMT2 #1, or shSHMT2 #2 were seeded in six-well plates at an initial density of 2 × 10^4^ cells per well. At the indicated time points, cells were harvested by trypsinization, stained with trypan blue, and counted using a cell counter. Cell growth curves were generated based on the number of viable cells. Each experiment was performed independently three times.

### 2.12. Cell Viability Assay

Cells were seeded in 96-well plates at a density of 2 × 10^3^ cells per well. After the indicated treatments, Cell Counting Kit-8 (CCK-8; Dojindo, Kumamoto, Japan) reagent was added and incubated at 37 °C. Cell viability was assessed at 0, 24, 48, and 72 h by measuring absorbance at 452 nm using a multimode microplate reader (FilterMax F5, Molecular Devices, San Jose, CA, USA). For rescue experiments, cells were pretreated with the SHMT1/2 inhibitor SHIN2 for 24 h and subsequently co-treated with Ferrostatin-1 (HY-100579, MedChemExpress, Monmouth Junction, NJ, USA), Necrostatin-1 (HY-15760 MedChemExpress, Monmouth Junction, NJ, USA), or Z-VAD-FMK (S7023-GRP, Universal Biologicals Ltd., Cambridge, UK) for an additional 48 h. SHMT2 knockdown cells were treated under the same conditions. Cell viability curves were generated based on optical density values. For experiments using an alternative ferroptosis inducer, control and *SHMT2*-knockdown SNU1041 and MSK-QLL1 cells were treated with RSL3 (200 nM) for 24 h. Cell viability was assessed by CCK-8 assay.

### 2.13. PI Staining and Cell Death Quantification

Cell death was assessed by propidium iodide (PI) staining followed by flow cytometric analysis. *SHMT2*-knockdown SNU1041 and MSK-QLL1 cells were harvested following the indicated treatments, stained with PI according to the manufacturer’s instructions, and analyzed by flow cytometry. The proportion of PI-positive cells was quantified as an indicator of loss of plasma membrane integrity. For ferroptosis rescue experiments, cells were treated with Liproxstatin-1 (Lip-1, 200 nM) or Deferoxamine (DFO, 100 μM) under the indicated conditions before PI staining and cell viability analysis.

### 2.14. GSH/GSSG Assay

Intracellular glutathione (GSH) and oxidized glutathione (GSSG) concentrations were determined using a GSH/GSSG assay kit (Solarbio Life Sciences, Beijing, China) following the manufacturer’s protocol. Briefly, harvested cells were lysed by freeze–thaw cycles in the provided extraction buffer and centrifuged to collect the supernatant. To measure total GSH, lysates were reacted with 5,5′-dithiobis-(2-nitrobenzoic acid) (DTNB) coupled with glutathione reductase to yield a yellow 5-thio-2-nitrobenzoic acid (TNB) product. For GSSG quantification, endogenous reduced GSH was pre-masked using a specific derivative reagent prior to the DTNB reaction. Absorbance was monitored at 412 nm with a multimode microplate reader (FilterMax F5, Molecular Devices, San Jose, CA, USA), and the GSH/GSSG ratio was calculated using absolute concentration standard curves. Each assay was independently repeated three times.

### 2.15. Lipid Peroxidation Assay

Lipid peroxidation was assessed using the C11-BODIPY 581/591 fluorescent probe (Invitrogen, USA). Cells were incubated with 10 μM probe for 30 min at 37 °C, followed by analysis using flow cytometry. Fluorescence signals were detected at 488/510 nm (oxidized) and 581/591 nm (reduced), and lipid peroxidation was quantified as the ratio of oxidized to reduced fluorescence intensity. Each experiment was performed independently in triplicate. For RSL3 experiments, control and *SHMT2*-knockdown cells were treated with RSL3 (200 nM) for 24 h before C11-BODIPY 581/591 staining and flow cytometric analysis.

### 2.16. Iron Assay

Intracellular ferrous iron (Fe^2+^) levels were quantified using a colorimetric Iron Assay Kit (ab83366; Abcam) according to the manufacturer’s instructions. Cells were harvested and homogenized in Iron Assay Buffer. For each reaction, 20 μL of sample was added to a 96-well plate and adjusted to a final volume of 100 μL with Iron Assay Buffer. For Fe^2+^ measurement, 5 μL of Iron Assay Buffer was added to each sample well, followed by incubation at 37 °C for 30 min. Subsequently, 100 μL of Iron Probe was added, and the samples were incubated at 37 °C for 60 min protected from light. Absorbance was measured at 593 nm. Fe^2+^ levels were calculated from a standard curve and normalized to 10^6^ cells.

### 2.17. Animal Experiments

Six-week-old female BALB/c nude mice weighing 17–19 g were purchased from Saeronbio (Uiwang, Gyeonggi-do, Republic of Korea) and maintained under specific pathogen-free (SPF) conditions at the Preclinical Animal Center of Chungnam National University College of Medicine. Mice were acclimatized for at least 7 days before experiments. Animals were housed four per cage in individually ventilated cages (IVC) under standard temperature (22 ± 2 °C), humidity (50 ± 10%), and a 12 h light/12 h dark cycle with free access to standard food and water. Environmental enrichment was provided. Animals were monitored regularly for general appearance, activity, food/water intake, body weight, tumor burden, and signs of pain or distress. All animal experiments were approved by the Institutional Animal Care and Use Committee of Chungnam National University Hospital (protocol No. CNUH-2024-IA0091-00; approved on 30 December 2024) and were not preregistered in a public repository. The experimental unit was an individual mouse (total n = 16; n = 4 per group). No formal a priori sample-size calculation was performed; group size was determined based on prior xenograft experience to minimize animal use, and no animals or data points were excluded from analysis.

Mice were subcutaneously injected with 1 × 10^7^ SNU1041 cells (shNC or shSHMT2) suspended in Matrigel. Once tumors were established, mice in each cohort were randomly allocated into vehicle- or erastin-treated groups (shNC + vehicle, shNC + erastin, shSHMT2 + vehicle, and shSHMT2 + erastin), although the specific randomization sequence was not recorded. Erastin (15 mg/kg) or vehicle (corn oil) was administered intraperitoneally every other day. No additional measures were used to control treatment/measurement order or cage location, and investigators were not blinded during treatment, outcome assessment, or data analysis. Tumor size and body weight were measured every two days using digital calipers and an electronic balance, respectively. A maximum tumor volume of 1500 mm^3^ was used as the humane endpoint; no animals reached this limit or required early euthanasia. Longitudinal tumor volume was designated as the primary outcome and calculated using the formula (length × width^2^)/2. At the experimental endpoint, mice were euthanized by CO_2_ inhalation and tumors were harvested.

### 2.18. Histological and Immunohistochemical Analysis

Tumor tissues were fixed in 4% paraformaldehyde, paraffin-embedded, and sectioned. Immunohistochemistry was performed following standard procedures with heat-mediated antigen retrieval. Sections were incubated overnight at 4 °C with primary antibodies against SHMT2 (1:500, #33443S, CST), GPX4 (1:500, 30388-1-AP, Proteintech, IL, USA), and 4-HNE (1:100, ab46545, Abcam), followed by HRP-conjugated secondary antibodies. Signals were visualized using DAB (Dako, Agilent, Santa Clara, CA, USA). Slides were counterstained with hematoxylin and scanned using an automated digital slide scanner (Pannoramic MIDI, 3DHISTECH, Budapest, Hungary).

### 2.19. Statistical Analysis

Unless otherwise indicated, quantitative data are presented as the mean ± SD. Statistical analyses were performed using GraphPad Prism version 9.5 (GraphPad Software). Two-group comparisons were conducted using unpaired two-tailed Student’s *t*-tests. Comparisons involving one experimental factor were performed using one-way ANOVA, whereas factorial experiments involving two independent factors were analyzed using two-way ANOVA. For multiple comparisons involving a single independent variable, ordinary one-way ANOVA followed by Šídák’s multiple-comparisons test was used to compare each experimental group with the corresponding control group. Interaction effects were also assessed in factorial analyses. Longitudinal tumor growth and body weight data were analyzed using repeated-measures two-way ANOVA followed by Šídák’s multiple-comparisons test. Paired clinical samples were analyzed using the Wilcoxon matched-pairs signed-rank test, and gene-expression correlations were assessed using Pearson’s correlation analysis. All statistical tests were two-sided. The number of independent biological replicates is indicated in the figure legends, and in vitro experiments were independently repeated at least three times. *p* < 0.05 was considered statistically significant.

## 3. Results

### 3.1. SHMT2 Suppression Reduces Cell Growth and Migration in Head and Neck Cancer Cells

**Figure 1:**
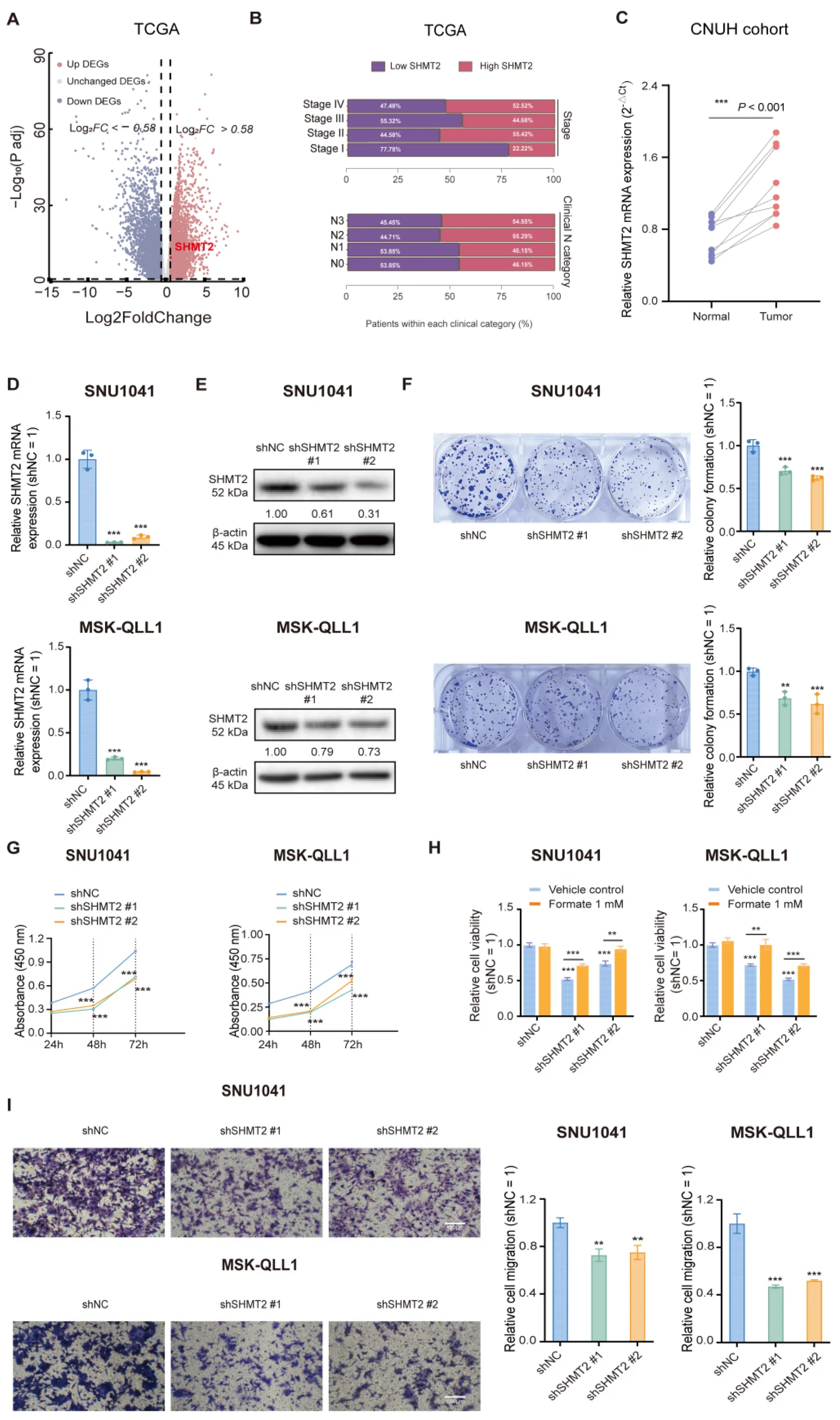
SHMT2 suppression reduces cell growth and migration in head and neck cancer cells. (**A**) Differentially expressed genes between the *SHMT2*-high and *SHMT2*-low groups were analyzed in the TCGA-HNSC cohort. Upregulated DEGs (red, log2FC > 0.58) and downregulated DEGs (blue, log2FC < −0.58) are indicated according to the corresponding cutoff lines. (**B**) Distribution of *SHMT2*-low and SHMT2-high tumors across clinical stage and clinical N categories in the TCGA-HNSC cohort. Unknown N status (NX) was excluded. Multivariable analyses are presented in Tables S3 and S4. (**C**) Relative *SHMT2* mRNA levels in paired tumor and adjacent normal tissues (*n* = 9) were quantified by qRT–PCR (2^−ΔΔCt^). Each line represents a matched sample. (**D**,**E**) SHMT2 knockdown efficiency was verified in SNU1041 and MSK-QLL1 cells transfected with non-targeting control shRNA (shNC) or *SHMT2*-targeting shRNAs (shSHMT2 #1 and shSHMT2 #2) by qRT–PCR and Western blotting, respectively. (**F**) Representative images and quantification of colony formation assays showing reduced colony-forming ability following SHMT2 silencing. Statistical comparisons were performed between each *SHMT2*-knockdown group and the shNC control. (**G**) CCK-8 assays assessing cell proliferation at the indicated time points following SHMT2 knockdown. (**H**) Formate supplementation (1 mM) partially restored cell viability following SHMT2 knockdown in SNU1041 and MSK-QLL1 cells. (**I**) Transwell assays evaluating the effect of SHMT2 depletion on cell migration. Unless otherwise indicated, data are presented as the mean ± SD from three independent biological experiments (*n* = 3). Statistical significance was assessed using the Wilcoxon matched-pairs signed-rank test (**C**), ordinary one-way ANOVA followed by Šídák’s multiple-comparisons test (**D**,**F**,**I**), or ordinary two-way ANOVA followed by Šídák’s multiple-comparisons test (**G**,**H**). ** *p* < 0.01, and *** *p* < 0.001.

Our previous study demonstrated that SHMT2 is highly expressed in HNSCC tissues and contributes to tumor progression [11]. To further investigate SHMT2-associated transcriptional alterations in HNSCC, we stratified TCGA-HNSC tumors into *SHMT2*-high and *SHMT2*-low groups and compared their gene expression profiles (Figure 1A). Figure 1B shows the unadjusted distribution of *SHMT2*-low and *SHMT2*-high tumors across clinical stage and clinical N categories. Complementary multivariable logistic regression analyses showed that the association between *SHMT2* expression and advanced clinical stage was not statistically significant after adjustment, whereas the association with clinical node positivity remained nominally significant (Tables S3 and S4). *SHMT2* expression was further validated in an independent cohort of paired clinical HNSCC samples from Chungnam National University Hospital (CNUH), showing significant upregulation in tumor tissues compared to adjacent normal tissues (*p* < 0.001) (Figure 1C). Furthermore, SNU1041 and MSK-QLL1 cells were transduced with two independent short hairpin RNAs (shRNAs) targeting *SHMT2* to assess its functional role in HNSCC. Efficient *SHMT2* knockdown at both the mRNA and protein levels was confirmed by quantitative RT–PCR and immunoblot analyses, respectively (Figure 1D,E). Following successful SHMT2 knockdown, clonogenic assays demonstrated a significant reduction in the colony-forming ability of both cell lines. This was characterized by a decrease in both colony number and size compared with the scrambled control (shNC) group (Figure 1F). Direct cell counting further confirmed that SHMT2 knockdown significantly reduced proliferation in both SNU1041 and MSK-QLL1 cells (Figure S1). Similarly, CCK-8–based growth curves demonstrated that SHMT2 knockdown significantly reduced cell proliferation at 24, 48, and 72 h compared with the shNC group (Figure 1G). Given the established role of SHMT2 in one-carbon metabolism and formate production, we next examined whether exogenous formate could rescue the growth defects induced by genetic or pharmacological suppression of SHMT2. Notably, sodium formate supplementation largely restored the proliferation capacity of *SHMT2*-silenced SNU1041 and MSK-QLL1 cells (Figure 1H), as well as cells treated with the SHMT1/2 inhibitor SHIN2 (Figure S1), indicating that impaired one-carbon flux contributes to the observed growth inhibition. In addition to reduced proliferation, SHMT2 knockdown significantly suppressed cell migration, as demonstrated by Transwell migration assays. The number of migrated cells was markedly decreased in shSHMT2 cells compared with the scrambled control group, suggesting that SHMT2 is required not only for sustaining tumor cell growth but also for maintaining the migratory and potentially metastatic phenotype of HNSCC cells (Figure 1I). Collectively, these findings establish SHMT2 as a critical regulator of proliferative and migratory capacities in HNSCC cells and highlight its essential role in sustaining the aggressive tumor phenotype.

### 3.2. SHMT2 Suppression Induces Ferroptosis While Partially Engaging Other Cell Death Pathways

**Figure 2:**
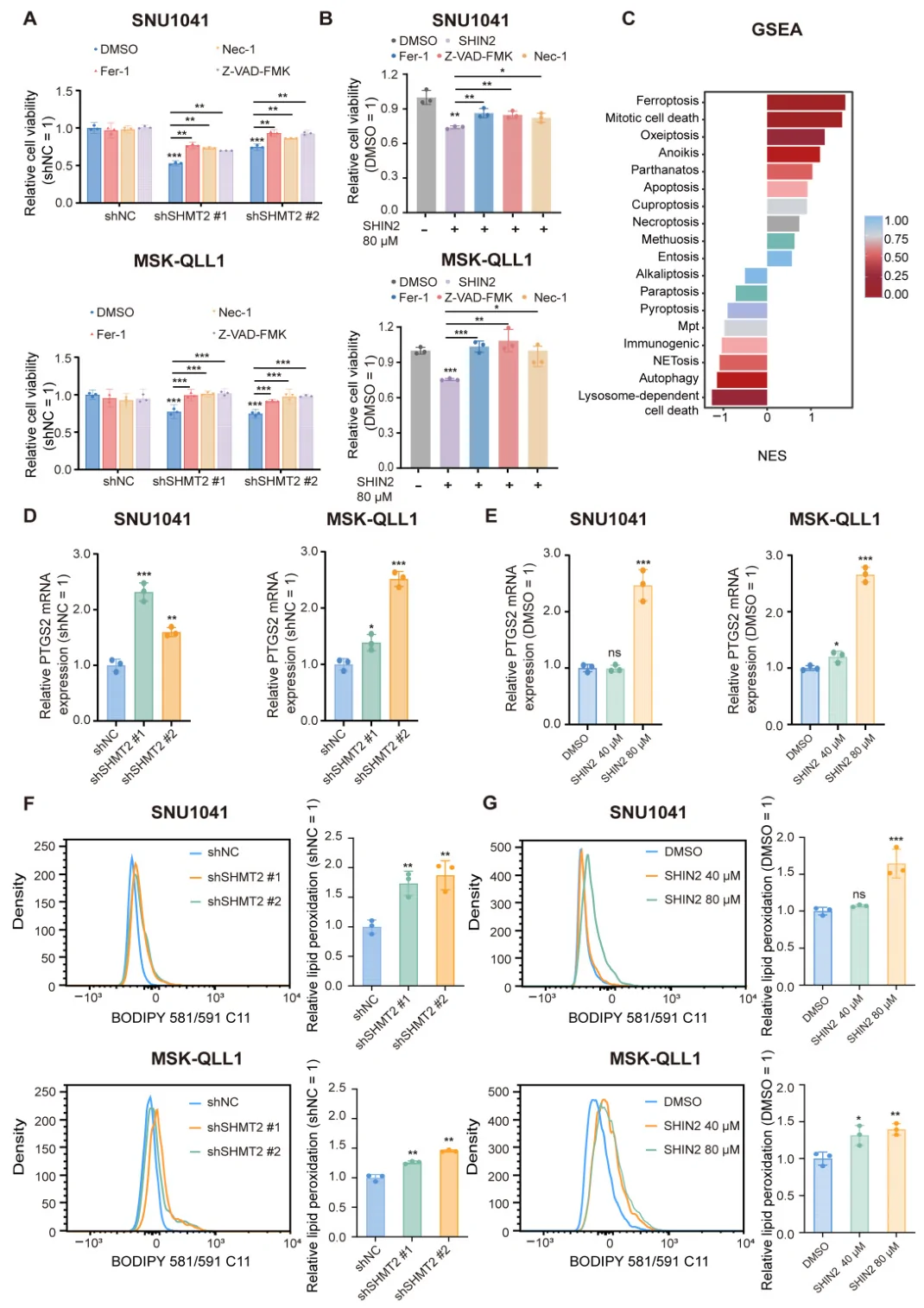
SHMT2 suppression induces ferroptosis while partially engaging other cell death pathways. (**A**,**B**) Cell viability was determined by CCK-8 assay in (**A**) *SHMT2*-silenced or (**B**) SHIN2-treated SNU1041 and MSK-QLL1 cells in the presence or absence of Ferrostatin-1 (Fer-1, 2 μM), Z-VAD-FMK (5 μM), or Necrostatin-1 (Nec-1, 5 μM) for 48 h. (**C**) GSEA plot showing enrichment of ferroptosis-related pathways in the low-SHMT2-expression group of the TCGA-HNSC cohort (*n* = 520 tumors). (**D**,**E**) *PTGS2* mRNA expression levels were analyzed by qRT–PCR in SNU1041 and MSK-QLL1 cells after *SHMT2* genetic knockdown (shNC, shSHMT2 #1, shSHMT2 #2) (**D**) or SHIN2 treatment (40 or 80 µM, 24 h) (**E**). (**F**,**G**) Lipid peroxidation was assessed by C11-BODIPY 581/591 staining and flow cytometry following SHMT2 knockdown (**F**) or SHIN2 treatment (**G**). Unless otherwise indicated, data are presented as the mean ± SD from three independent biological experiments (*n* = 3). Statistical significance was assessed using ordinary two-way ANOVA followed by Šídák’s multiple-comparisons test (**A**) or ordinary one-way ANOVA followed by Šídák’s multiple-comparisons test (**B**,**D**–**G**). Statistical comparisons are indicated by lines. ns, not significant; * *p* < 0.05, ** *p* < 0.01, and *** *p* < 0.001.

To complement our genetic knockdown studies and evaluate the impact of pharmacological inhibition of the SHMT axis, HNSCC cells were treated with SHIN2, a dual SHMT1/2 inhibitor [27]. Both approaches markedly reduced cell viability. Rescue experiments using inhibitors targeting distinct cell death pathways revealed that Ferrostatin-1 (Fer-1), Necrostatin-1 (Nec-1), and Z-VAD-FMK each partially restored cell viability to varying extents, suggesting the involvement of multiple cell death programs (Figure 2A,B). However, because subsequent transcriptomic analyses and ferroptosis-related molecular assessments consistently supported ferroptosis activation (Figure 2C), we focused on elucidating the role of SHMT2 in ferroptosis and its underlying molecular mechanisms. To further confirm ferroptosis induction upon SHMT2 suppression, we assessed prostaglandin-endoperoxide synthase 2 (PTGS2) expression, a commonly used transcriptional marker of ferroptosis [18]. SHMT2 suppression significantly increased *PTGS2* mRNA levels (Figure 2D,E). Importantly, SHMT2 suppression markedly increased lipid peroxidation, as determined by C11-BODIPY 581/591 staining and flow cytometric analysis (Figure 2F,G). To evaluate the contribution of ferroptotic cell death following SHMT2 suppression, membrane permeability loss was directly quantified using propidium iodide (PI) staining and flow cytometry. Genetic depletion of SHMT2 markedly increased the proportion of PI-positive cells in both SNU1041 and MSK-QLL1 cells. Treatment with the ferroptosis inhibitor Liproxstatin-1 significantly reduced the increase in PI-positive cells and partially restored cell viability, providing additional pharmacological evidence supporting the involvement of ferroptotic cell death (Figure S2A,B). In contrast, the iron chelator Deferoxamine did not significantly rescue SHMT2 knockdown-induced cell death or loss of viability under the conditions examined (Figure S2C,D). Intracellular Fe^2+^ levels were comparable between control and *SHMT2*-knockdown SNU1041 and MSK-QLL1 cells, ranging from 8.0 to 9.3 nmol/10^6^ cells (Figure S2E). These findings indicate that SHMT2 depletion did not significantly alter the measured intracellular Fe^2+^ levels under the conditions examined. Taken together, these findings further support the contribution of ferroptosis to SHMT2 suppression-induced cell death, while also indicating that ferroptosis is unlikely to represent the sole mechanism involved.

### 3.3. SHMT2 Suppression Downregulates GPX4 and Ferroptosis Defense Components

**Figure 3:**
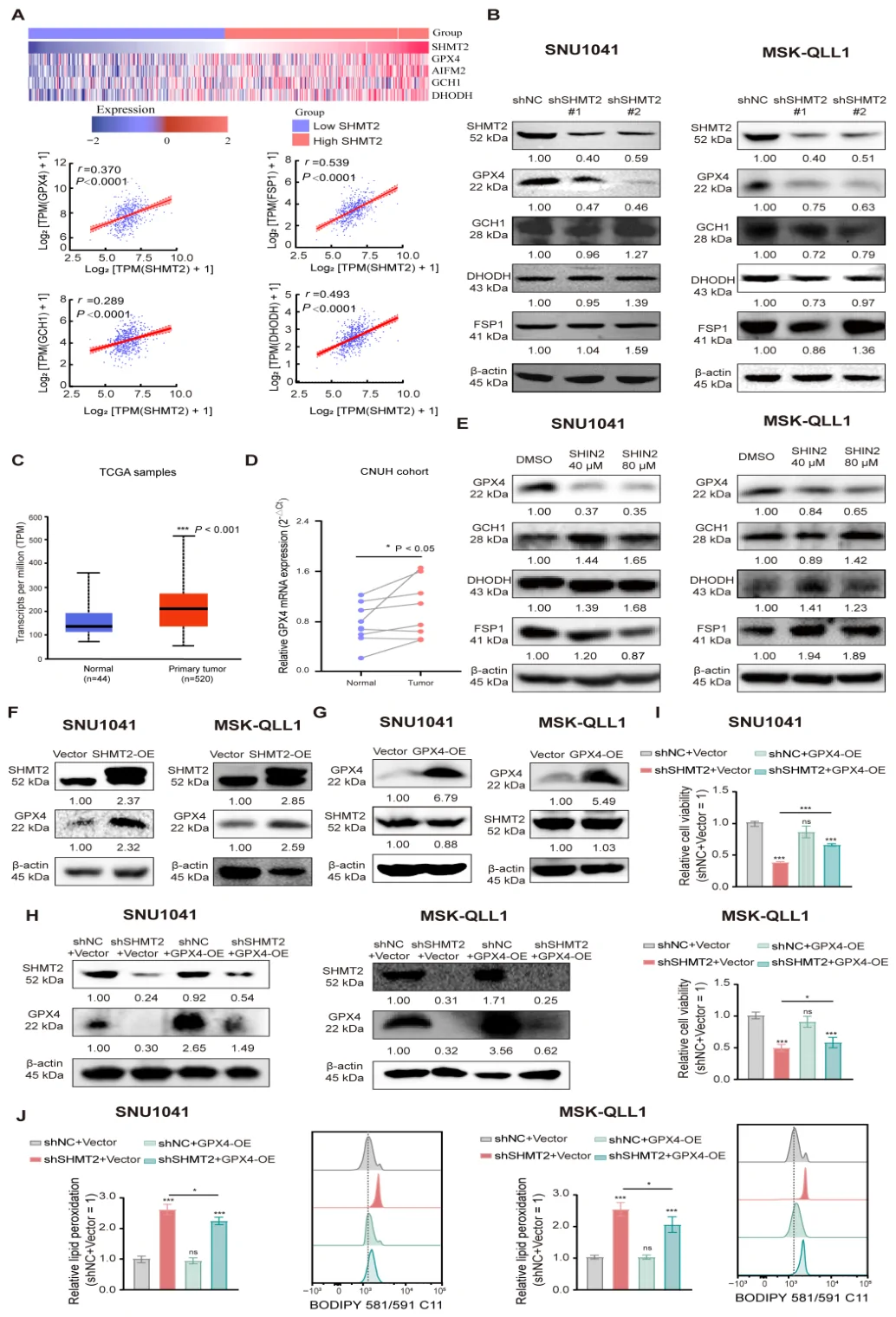
SHMT2 suppression downregulates GPX4 and ferroptosis defense components. (**A**) Expression and correlation analyses of ferroptosis-protective genes (*GPX4 GCH1*, *DHODH*, and *FSP1*) according to *SHMT2* expression in the TCGA-HNSC cohort. The heatmap (**top**) shows the expression patterns of ferroptosis-protective genes in *SHMT2*-low and *SHMT2*-high tumors. The scatter plots (**bottom**) demonstrate positive correlations between SHMT2 and each ferroptosis-protective gene. (**B**) Western blot analysis of SHMT2, GPX4, GCH1, DHODH, and FSP1 expression in SNU1041 and MSK-QLL1 cells following SHMT2 knockdown. (**C**) GPX4 transcript levels in normal tissues (*n* = 44) and primary HNSCC tissues (*n* = 520) were analyzed using the UALCAN portal based on the TCGA-HNSC dataset and are presented as transcripts per million (TPM). (**D**) Relative *GPX4* mRNA levels were quantified by qRT–PCR (2^−ΔΔCt^) in paired tumor and adjacent normal tissues from the CNUH cohort (*n* = 9). Each line represents a matched sample. Statistical significance was determined using the Wilcoxon matched-pairs signed-rank test. (**E**) Western blot analysis of GPX4, GCH1, DHODH, and FSP1 expression in SNU1041 and MSK-QLL1 cells following SHIN2 treatment (40–80 μM, 24 h). (**F**) Western blot analysis showed that *SHMT2* overexpression increased GPX4 protein levels in both SNU1041 and MSK-QLL1 cells. (**G**) Conversely, *GPX4* overexpression did not appreciably affect SHMT2 protein levels in either cell line. (**H**) Western blot analysis of SHMT2 and GPX4 in control (shNC) and *SHMT2*-knockdown (shSHMT2) SNU1041 and MSK-QLL1 cells transfected with an empty vector or a *GPX4* overexpression plasmid. β-Actin was used as a loading control. (**I**) Relative cell viability in the four indicated groups in SNU1041 and MSK-QLL1 cells. (**J**) Quantification of C11-BODIPY 581/591 staining and representative flow cytometry histograms showing lipid peroxidation in the four indicated groups in SNU1041 and MSK-QLL1 cells. Representative immunoblots from three independent experiments with similar results are shown in panels *(***B**,**E***–***H**); displayed band intensities were quantified using ImageJ and normalized to β-actin. Pearson correlation analysis was used for panel (**A**), UALCAN-provided statistics for panel (**C**), and the Wilcoxon matched-pairs signed-rank test for panel (**D**). Panels (**I**,**J**) were analyzed using ordinary two-way ANOVA followed by Šídák’s multiple-comparisons test. ns, not significant; * *p* < 0.05 and *** *p* < 0.001.

To assess the association between *SHMT2* expression and key ferroptosis-protective genes, we analyzed four representative genes, *GPX4*, *GCH1*, *DHODH*, and *FSP1* [18,28] using the TCGA-HNSC cohort. Tumors were stratified into *SHMT2*-high and *SHMT2*-low groups, and all four genes showed varying degrees of upregulation in the *SHMT2*-high group (Figure 3A). Correlation analysis further revealed positive associations between *SHMT2* and each of these genes across tumor samples (Figure 3A). These tumor-level co-expression patterns indicate associations and do not establish regulatory direction or causality. To further determine whether SHMT2 suppression affects ferroptosis-defense components, their protein expression was examined by Western blotting. In both SNU1041 and MSK-QLL1 cells, genetic depletion of SHMT2 markedly reduced GPX4 protein abundance, whereas GCH1, DHODH, and FSP1 showed inconsistent changes between cell lines (Figure 3B). We next examined whether GPX4 was also dysregulated in HNSCC tissues. *GPX4* mRNA expression was significantly increased in primary tumor tissues compared with normal tissues in the TCGA-HNSC dataset (*p* < 0.001) (Figure 3C). Consistently, GPX4 expression was elevated in paired clinical HNSCC samples from the CNUH cohort (Figure 3D), paralleling the upregulation of SHMT2 observed in the same patient group (Figure 1C). Pharmacological SHMT2 inhibition with SHIN2 also decreased GPX4 expression, whereas the changes in GCH1, DHODH, and FSP1 were less consistent (Figure 3E). Among the ferroptosis-protective genes examined, GPX4 showed the most consistent response to SHMT2 suppression and was therefore selected for further validation. To determine whether SHMT2 regulates GPX4 at the transcriptional or post-transcriptional level, *GPX4* mRNA expression was evaluated by qRT-PCR following SHMT2 knockdown or SHIN2 treatment. Neither genetic depletion nor pharmacological inhibition of SHMT2 significantly altered *GPX4* mRNA levels in SNU1041 and MSK-QLL1 cells (Figure S3C,D). In contrast, GPX4 protein levels were reduced under both conditions (Figure S3A,B), suggesting that SHMT2 suppression affects GPX4 predominantly through a post-transcriptional mechanism rather than by altering *GPX4* mRNA expression. Given the central role of GPX4 in limiting lipid peroxidation, we next examined components of the GPX4-associated antioxidant pathway following SHMT2 suppression. Because xCT/SLC7A11 is an upstream component of the GSH–GPX4 axis [29,30], its expression was also evaluated following SHMT2 suppression. However, xCT expression showed cell line–dependent alterations, suggesting that xCT regulation is not a consistent downstream event of SHMT2 suppression (Figure S3A,B). To further confirm the association between SHMT2 and GPX4 expression, GPX4 protein levels were assessed under *SHMT2*-overexpressing conditions. Notably, ectopic re-expression of SHMT2 restored GPX4 levels, further supporting a regulatory link between SHMT2 and GPX4 expression (Figure 3F). Conversely, *GPX4* overexpression had no appreciable effect on SHMT2 protein levels (Figure 3G), further supporting a directional relationship in which SHMT2 functions upstream of GPX4. To test whether GPX4 functionally mediates the effects of SHMT2 suppression, we overexpressed GPX4 in *SHMT2*-knockdown SNU1041 and MSK-QLL1 cells. Immunoblotting confirmed increased GPX4 expression following transfection (Figure 3H). *GPX4* overexpression partially restored cell viability (Figure 3I) and reduced lipid peroxidation (Figure 3J) in *SHMT2*-knockdown cells. These results support a functional role for GPX4 downstream of SHMT2.

### 3.4. SHMT2 Suppression Induces Ferroptosis by Disrupting Glutathione Redox Balance

**Figure 4:**
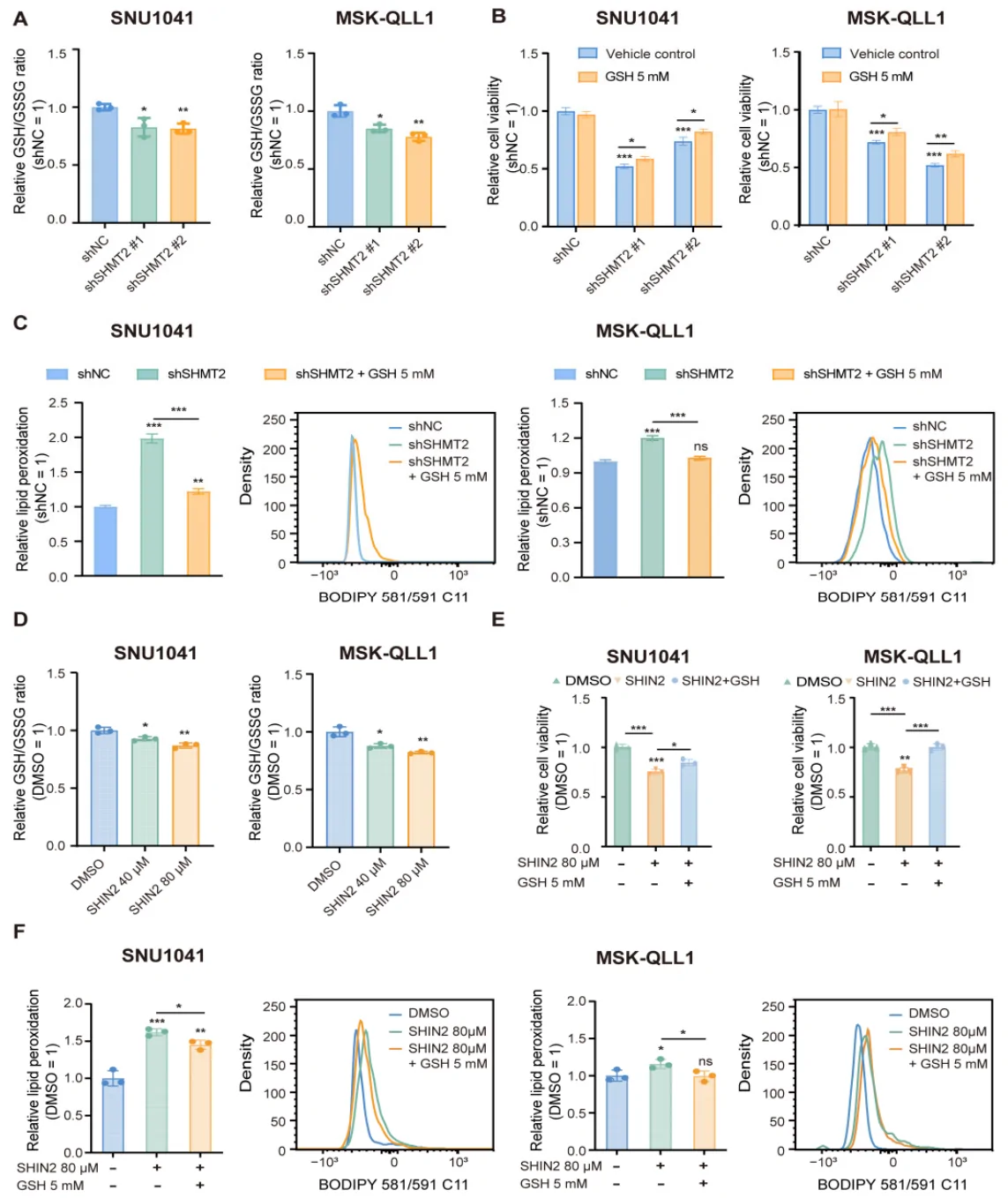
*SHMT2* suppression induces ferroptosis by disrupting glutathione redox balance. (**A**) The GSH/GSSG ratio was decreased in *SHMT2*-knockdown SNU1041 and MSK-QLL1 cells. (**B**,**C**) Exogenous GSH (5 mM) restored cell viability and reduced lipid peroxidation in *SHMT2*-depleted cells. (**D**–**F**) Pharmacological SHMT2 inhibition with SHIN2 (40 or 80 μM, 24 h) decreased the GSH/GSSG ratio and induced ferroptotic stress, whereas GSH supplementation partially restored cell viability and reduced lipid peroxidation. Unless otherwise indicated, data are presented as the mean ± SD from three independent biological experiments (*n* = 3). Panels (**A**–**F**) were analyzed using ordinary one-way ANOVA followed by Šídák’s multiple-comparisons test. Statistical comparisons are indicated by lines. ns, not significant; * *p* < 0.05, ** *p* < 0.01, and *** *p* < 0.001.

To assess intracellular redox homeostasis following *SHMT2* suppression, we measured the GSH/GSSG ratio. *SHMT2* suppression significantly decreased the GSH/GSSG ratio in both SNU1041 and MSK-QLL1 cells, indicating disruption of cellular redox balance (Figure 4A). Because GPX4 requires GSH as an essential cofactor for detoxifying lipid hydroperoxides, the effect of GSH restoration on lipid peroxidation and cell viability in *SHMT2*-knockdown cells was subsequently assessed. Supplementation with exogenous GSH markedly improved the viability of *SHMT2*-knockdown SNU1041 and MSK-QLL1 cells (Figure 4B). In addition, GSH supplementation significantly attenuated SHMT2 depletion-induced lipid peroxidation, as determined by flow cytometric analysis (Figure 4C). We next evaluated the effect of pharmacological SHMT2 suppression on redox homeostasis. Treatment with the SHMT1/2 inhibitor SHIN2 resulted in a dose-dependent decrease in the GSH/GSSG ratio (Figure 4D). Exogenous GSH substantially reversed SHIN2-induced loss of cell viability in both cell lines (Figure 4E). Moreover, GSH supplementation effectively suppressed the increase in lipid peroxidation triggered by SHIN2 treatment (Figure 4F). Together, these findings indicate that SHMT2 suppression disrupts intracellular redox balance by reducing the GSH/GSSG ratio, thereby impairing GPX4-dependent detoxification of lipid peroxides and predisposing HNSCC cells to ferroptotic stress.

### 3.5. SHMT2 Suppression Enhances Erastin-Induced Ferroptosis

**Figure 5:**
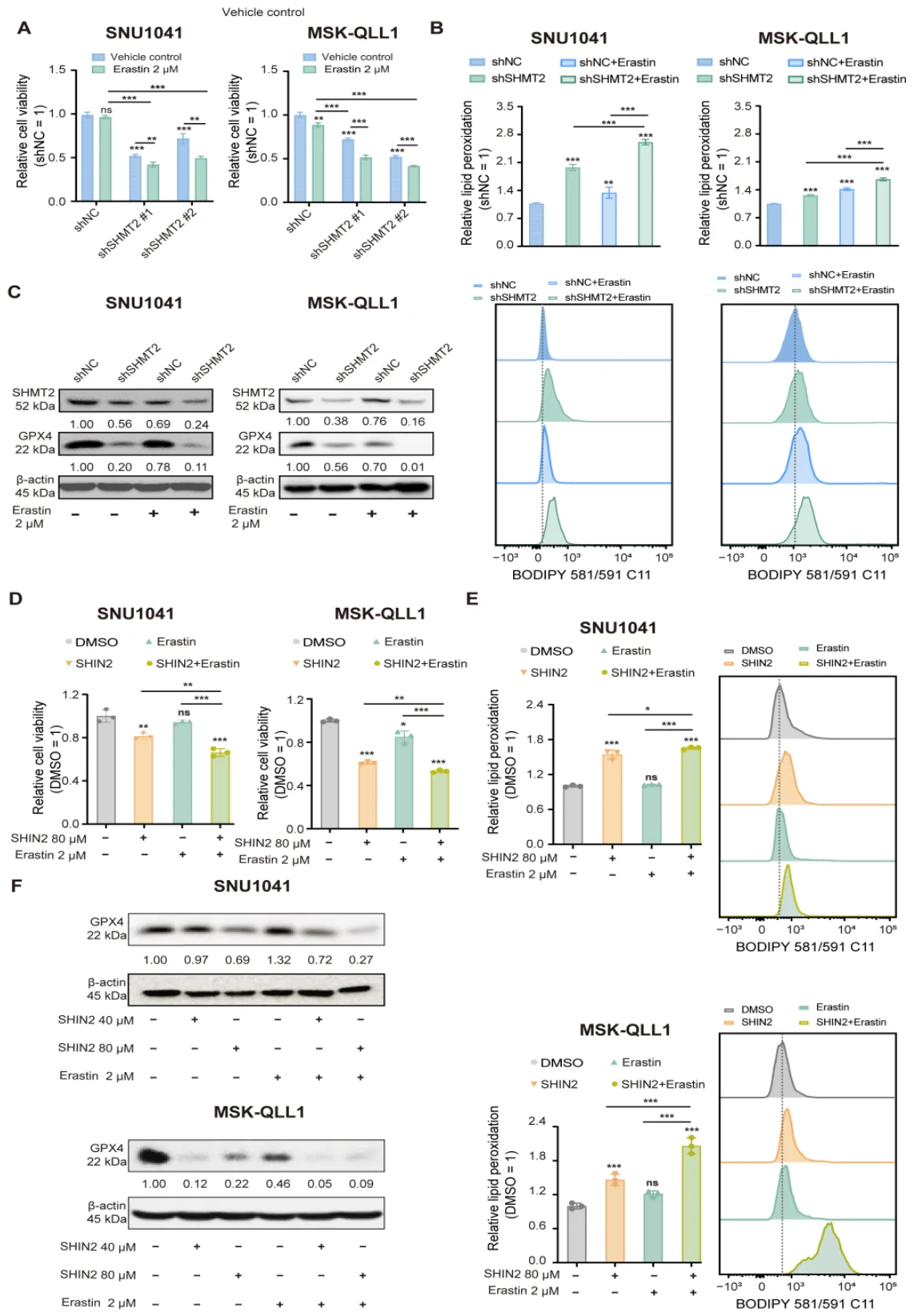
*SHMT2* suppression enhances erastin-induced ferroptosis. (**A**,**B**) SHMT2 knockdown sensitized SNU1041 and MSK-QLL1 cells to erastin (2 μM), resulting in decreased cell viability and increased lipid peroxidation. (**C**) Western blot analysis showing reduced GPX4 protein levels in *SHMT2*-depleted cells treated with erastin. (**D**,**E**) Pharmacological SHMT2 inhibition with SHIN2 (80 μM) enhanced erastin-induced ferroptotic cell death and lipid peroxidation. (**F**) Western blot analysis showing that combined SHIN2 and erastin treatment further decreased GPX4 protein expression compared with single-agent treatment. Data are presented as the mean ± SD from three independent biological experiments (*n* = 3). Panels (**A**,**B**,**D**,**E**) were analyzed using ordinary two-way ANOVA followed by Šídák’s multiple-comparisons test. Representative immunoblots from three independent experiments are shown in panels (**C**,**F**). Statistical comparisons are indicated by lines. ns, not significant; * *p* < 0.05, ** *p* < 0.01, and *** *p* < 0.001.

Erastin is one of the earliest and most widely used pharmacological inducers of ferroptosis and exerts its effects primarily by inhibiting the cystine/glutamate antiporter System xCT, thereby disrupting the xCT–GSH–GPX4 antioxidant axis [14]. In this pathway, cystine imported via xCT together with glycine generated through SHMT2-mediated serine metabolism and glutamate is required for glutathione (GSH) synthesis, suggesting a metabolic coordination between SHMT2-dependent glycine metabolism and cystine availability in regulating GPX4-dependent antioxidant defense [30,31]. To determine whether SHMT2 loss sensitizes cells to ferroptosis, we first established sublethal concentrations of erastin that minimally affected cell viability. As shown in Figure S4, erastin treatment at 1–4 μM induced little or no cytotoxicity and was therefore used as a functional probe to evaluate *SHMT2*-dependent ferroptosis sensitivity. Given the established role of SHMT2 in supporting GPX4-dependent ferroptosis resistance, the impact of SHMT2 loss on cellular responses to erastin was subsequently evaluated. Under erastin treatment, *SHMT2*-deficient SNU1041 and MSK-QLL1 cells exhibited significantly lower cell viability compared with control cells (Figure 5A). Consistent with these observations, erastin-induced lipid peroxidation was significantly elevated in *SHMT2*-knockdown cells (Figure 5B), revealing that SHMT2 depletion increases ferroptotic susceptibility. Erastin further reduced GPX4 protein expression, and this effect was more pronounced in *SHMT2*-silenced cells (Figure 5C), further supporting the notion that SHMT2 suppression enhances cellular sensitivity to erastin-induced ferroptotic stress. To further validate this enhanced effect, the SHMT1/2 inhibitor SHIN2 was combined with erastin. Co-treatment corroborated a greater loss of cell viability, higher lipid peroxidation, and further downregulation of GPX4 relative to erastin alone (Figure 5D–F). Collectively, these findings support the notion that SHMT2 suppression heightens cellular vulnerability to erastin and that dual targeting of SHMT2 and ferroptosis exerts enhanced antitumor effects. To further determine whether SHMT2 suppression enhances susceptibility to an alternative ferroptosis inducer, control and *SHMT2*-knockdown cells were treated with the GPX4 inhibitor RSL3 (200 nM) for 24 h. SHMT2 knockdown significantly enhanced RSL3-induced loss of cell viability and increased lipid peroxidation in both SNU1041 and MSK-QLL1 cells (Figure S4B,C), further supporting increased ferroptotic susceptibility following SHMT2 suppression.

### 3.6. SHMT2 Suppression Promotes Ferroptosis and Enhances the Antitumor Effect of Erastin In Vivo

**Figure 6:**
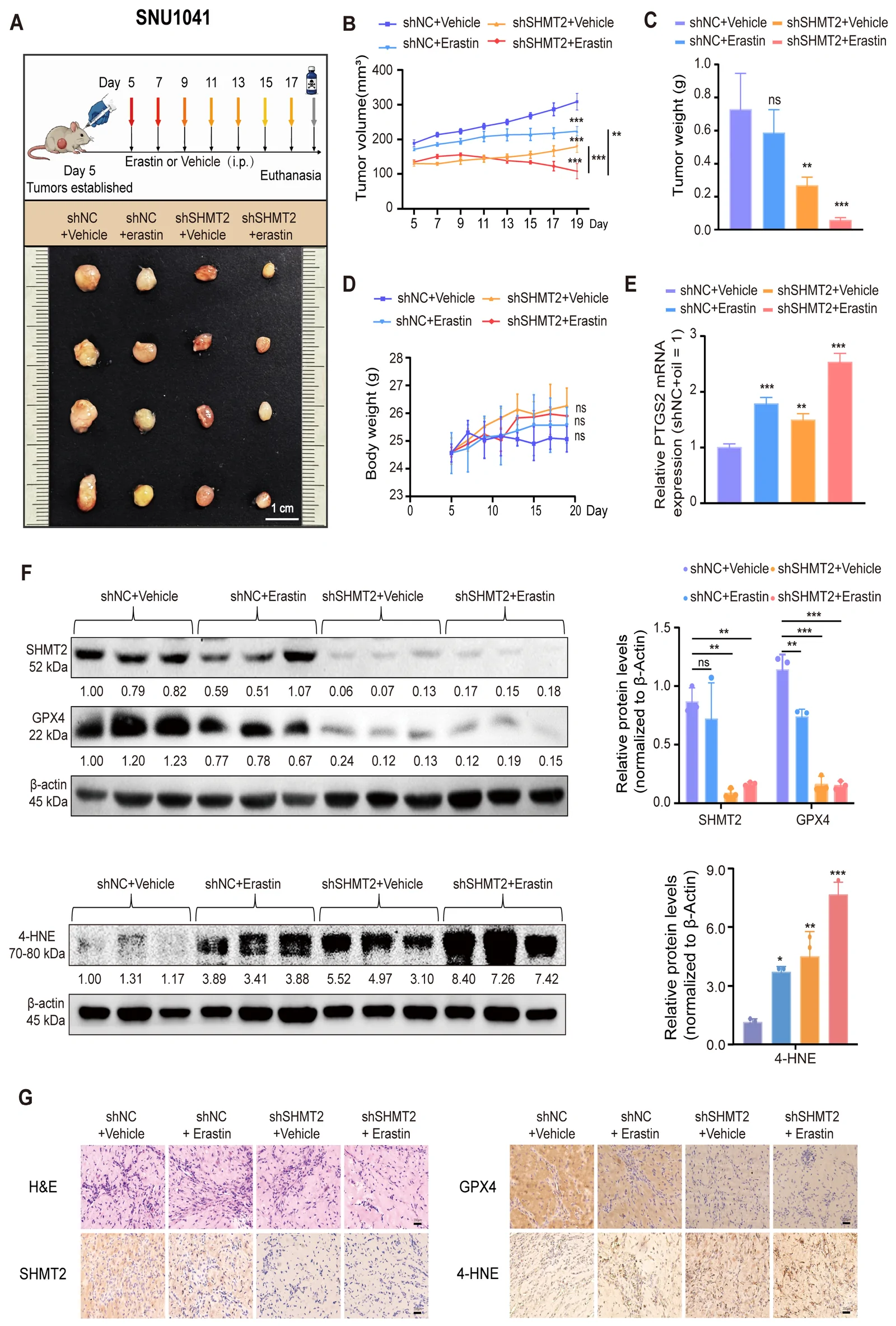
*SHMT2* suppression promotes ferroptosis and enhances the antitumor effect of erastin in vivo. (**A**) Treatment schedule and representative images of excised tumors from nude mice bearing SNU1041 xenografts under the indicated treatments. (**B**) Tumor growth curves showing that SHMT2 knockdown and erastin treatment each inhibited tumor growth, with the combination producing the greatest suppression. (**C**) Final tumor weights at endpoint. (**D**) Body weight changes during treatment, showing no significant differences among the groups. (**E**) *PTGS2* mRNA expression levels were analyzed by qRT–PCR in tumor tissues, showing increased expression in *SHMT2*-deficient tumors, which was further elevated upon erastin treatment. (**F**) Immunoblot analysis of SHMT2, GPX4, and 4-HNE in tumor tissues. GPX4 protein levels were decreased, whereas 4-HNE levels were increased, most prominently in the combination group. (**G**) Histological and immunohistochemical analyses (H&E, SHMT2, GPX4, and 4-HNE) revealed decreased GPX4 expression and increased 4-HNE accumulation in *SHMT2*-deficient tumors, with the most pronounced changes observed in the combination (shSHMT2 + erastin) group. Data are presented as the mean ± SD (*n* = 4 mice per group). Panels (**B**,**D**) were analyzed using repeated-measures two-way ANOVA (group × time), whereas panels (**C**,**E**) were analyzed using ordinary two-way ANOVA (shRNA × erastin), followed by Šídák’s multiple-comparisons test. Panels (**A**,**F**,**G**) show representative images only. ns, not significant; * *p* < 0.05, ** *p* < 0.01, and *** *p* < 0.001.

To validate whether SHMT2 suppression enhances ferroptosis and the antitumor effect of erastin in vivo, SNU1041 xenograft models were established. SNU1041 cells expressing either control shRNA (shNC) or SHMT2-targeting shRNA were subcutaneously implanted into nude mice. Mice were treated with erastin at 15 mg/kg by intraperitoneal injection (i.p.), and tumor growth was subsequently assessed. The treatment schedule and representative images of excised tumors are shown in Figure 6A. Tumor growth was monitored longitudinally. Both SHMT2 knockdown and erastin treatment significantly suppressed tumor progression relative to the control group, whereas the combination group (shSHMT2 + erastin) exhibited the most pronounced inhibition of tumor volume over time (Figure 6B). Consistent with these findings, endpoint tumor weights were markedly diminished in the combination group relative to either monotherapy (Figure 6C), indicating that SHMT2 suppression enhances the antitumor effect of erastin in vivo. Throughout the treatment period, body weights remained stable across all groups, and no statistically significant differences were observed, suggesting no overt treatment-related toxicity based on body-weight changes (Figure 6D). To assess whether enhanced tumor inhibition was accompanied by ferroptosis-associated molecular alterations, tumors were subjected to molecular characterization. Consistent with enhanced ferroptosis, *PTGS2* mRNA expression was significantly elevated in *SHMT2*-deficient tumors and further augmented following erastin treatment (Figure 6E). Immunoblot analysis confirmed efficient SHMT2 knockdown and demonstrated that GPX4 expression was markedly reduced following SHMT2 suppression and remained suppressed in the combination (shSHMT2 + erastin) group, whereas 4-HNE, a well-established marker of lipid peroxidation, exhibited the opposite pattern and was further elevated under combination treatment (Figure 6F). Histological (H&E) examination confirmed the presence of tumor tissues in all groups, whereas immunohistochemical staining revealed decreased GPX4 expression and increased 4-HNE accumulation in *SHMT2*-deficient tumors, with the most pronounced changes observed in the combination group (Figure 6G).

### 3.7. Proposed Working Model Illustrating How SHMT2 Suppression Sensitizes HNSCC Cells to Erastin-Induced Ferroptosis

**Figure 7:**
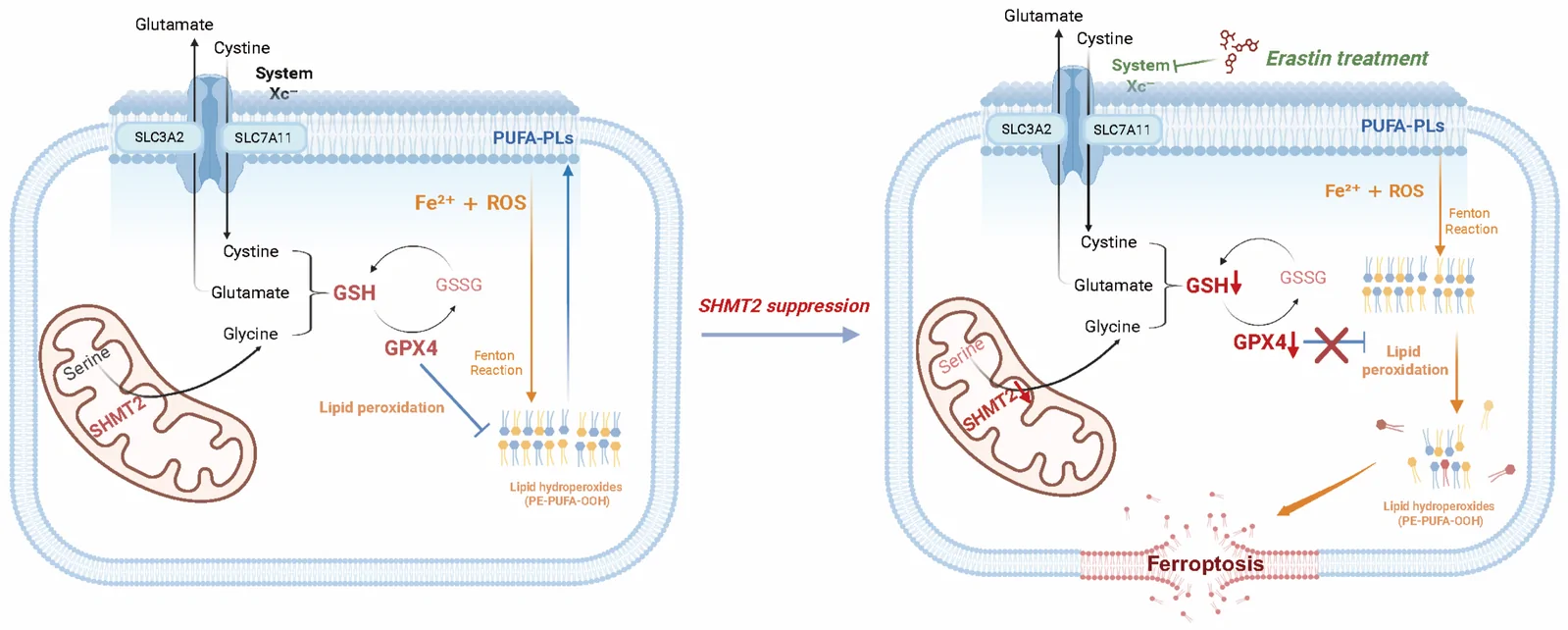
Proposed working model illustrating how *SHMT2* suppression sensitizes HNSCC cells to erastin-induced ferroptosis. Arrows connecting metabolites and proteins indicate the direction of molecular transport or metabolic reactions. The central arrow indicates the changes induced by SHMT2 suppression, while upward and downward arrows in the right panel denote increases and decreases, respectively. Created in BioRender. Erin, S. (2026) https://BioRender.com/re7ixe2, accessed on 15 December 2025.

Collectively, these findings suggest that SHMT2 suppression impairs the GSH–GPX4 ferroptosis-defense axis, promotes lipid peroxidation, and enhances tumor sensitivity to erastin-induced ferroptosis in vivo (Figure 7).

SHMT2 suppression impairs the GSH–GPX4 antioxidant defense axis, resulting in increased lipid peroxidation. Erastin further blocks cystine uptake mediated by system xc^−^, thereby reducing GSH synthesis and enhancing ferroptotic cell death. Together, SHMT2 suppression and erastin treatment cooperatively weaken ferroptosis defense mechanisms and promote lipid peroxide accumulation.

## 4. Discussion

For patients with unresectable, locoregionally advanced HNSCC, definitive chemoradiotherapy is a cornerstone of treatment. Nevertheless, intrinsic and acquired resistance remain major drivers of therapeutic failure and poor clinical outcomes [32]. In this context, ferroptosis, an iron-dependent form of regulated cell death, has attracted considerable attention in cancer biology. By disrupting cellular redox homeostasis, ferroptosis is increasingly recognized as a promising mechanism underlying therapeutic response and acquired resistance [14,16,22]. Importantly, the present study provides mechanistic insights into *SHMT2*-mediated ferroptosis regulation in conventional HNSCC models.

SHMT2, a key mitochondrial enzyme in one-carbon metabolism, plays important roles in nucleotide biosynthesis and redox regulation, thereby supporting tumor growth and metabolic adaptation. Our previous study demonstrated persistent overexpression of *SHMT2* in head and neck cancer and established its oncogenic role in promoting tumor progression [11]. In the present study, we used hypopharyngeal- and oral squamous cell carcinoma–derived cell lines and demonstrated that SHMT2 knockdown significantly suppressed proliferation in both models, suggesting that SHMT2 broadly contributes to HNSCC growth.

Accumulating evidence links *SHMT2* deficiency to intracellular ROS accumulation and oxidative stress across multiple tumor types. Ye et al. reported that *SHMT2* is induced by HIF-1 under hypoxia and promotes mitochondrial one-carbon metabolism–mediated NADPH production to maintain redox homeostasis [33]. Similarly, Zhang et al. demonstrated that *SHMT2* maintains redox homeostasis and protects bladder cancer cells from ROS-dependent, mitochondria-mediated apoptosis [13]. *SHMT2* depletion has also been shown to induce apoptosis through lysosomal membrane permeabilization (LMP) caused by excessive activation of the autophagy–lysosome pathway in clear cell renal cell carcinoma [12]. Collectively, these findings suggest that *SHMT2* functions as a central regulator of metabolic and redox homeostasis, thereby influencing tumor adaptation to oxidative stress and cell death pathways.

Importantly, our study extends the role of *SHMT2* from a general regulator of redox balance to a determinant of ferroptosis sensitivity in HNSCC. Du et al. (2024) first demonstrated that mitochondrial *SHMT2* suppresses ferroptosis in hematopoietic stem cells by maintaining redox homeostasis and preserving stem cell regenerative capacity [21]. Building upon these observations, our study provides evidence in a tumor context that *SHMT2* regulates ferroptotic vulnerability through modulation of the GSH–GPX4 lipid peroxide defense system. Together, these findings position *SHMT2* as a metabolic hub linking one-carbon metabolism, redox homeostasis, and ferroptosis regulation.

Mechanistically, our findings suggest that *SHMT2*-mediated ferroptosis regulation is not entirely equivalent to the canonical xCT-centered ferroptosis pathway. Erastin is a well-established ferroptosis inducer that primarily acts through inhibition of the xCT–GSH–GPX4 axis [14]. In contrast, xCT expression changes following *SHMT2* suppression were variable and cell line–dependent, indicating that *SHMT2*-mediated ferroptosis cannot be fully explained by xCT suppression alone. Instead, our data indicate that *SHMT2* sustains intracellular GSH availability and GPX4-dependent antioxidant capacity through serine–glycine metabolism. *SHMT2* suppression decreased the GSH/GSSG ratio, increased lipid peroxidation, and reduced GPX4 protein levels, whereas GSH supplementation partially restored cell viability and attenuated lipid peroxidation. The decreased GSH/GSSG ratio following *SHMT2* suppression should be interpreted as evidence of disrupted redox homeostasis rather than direct evidence of glycine-limited GSH synthesis. *SHMT2* may contribute to glutathione homeostasis by generating glycine, while mitochondrial serine catabolism through the SHMT2–MTHFD2 pathway can also support NAD(P)H production and antioxidant defense. Indeed, Du et al. demonstrated that mitochondrial serine catabolism through the SHMT2–MTHFD2 pathway supported NAD(P)H generation and limited ferroptosis susceptibility in hematopoietic stem cells [21]. However, the present study did not directly measure glycine flux, de novo GSH synthesis, or NADPH availability. Therefore, the observed decrease in the GSH/GSSG ratio may reflect reduced GSH synthesis, impaired GSSG recycling, increased oxidative consumption of GSH, or a combination of these processes. Future isotope-tracing and metabolic-flux analyses, together with direct measurement of NADPH levels, will be required to determine their relative contributions in HNSCC cells. *SHMT2* overexpression increased GPX4 abundance, while *GPX4* overexpression had little effect on SHMT2, placing SHMT2 upstream of GPX4. Importantly, functional rescue experiments showed that *GPX4* overexpression partially restored cell viability and reduced lipid peroxidation in *SHMT2*-knockdown SNU1041 and MSK-QLL1 cells. These findings further support a functional role for GPX4 downstream of SHMT2 in ferroptosis regulation. Notably, *GPX4* mRNA levels were not significantly altered following either genetic or pharmacological suppression of SHMT2, despite a clear reduction in GPX4 protein abundance. These findings suggest that SHMT2-associated regulation of GPX4 occurs predominantly at the post-transcriptional level rather than through changes in GPX4 transcription. These findings indicate that *SHMT2* supports GPX4-dependent antioxidant defense and may regulate GPX4 through mechanisms beyond its established role in serine–glycine metabolism. Although *SHMT2* suppression and erastin act through distinct upstream mechanisms, both converge on disruption of the GSH–GPX4 antioxidant axis. Consistent with the erastin findings, SHMT2 depletion also enhanced RSL3-induced loss of cell viability and lipid peroxidation, supporting increased susceptibility to ferroptotic stress induced through a distinct pharmacological mechanism. Rescue assays indicated that SHMT2 depletion may engage multiple cell-death pathways, while the additional ferroptosis-focused assays further support a contribution of ferroptosis to SHMT2 suppression-induced cell death. While genetic knockdown using shRNA specifically targeted SHMT2, pharmacological treatment with SHIN2, a dual SHMT1/2 inhibitor, produced concordant effects and therefore provides complementary evidence for the biological relevance of SHMT inhibition in HNSCC [27]. Collectively, these findings establish *SHMT2* as a regulator of ferroptosis sensitivity through maintenance of the GSH–GPX4 antioxidant system.

From a translational perspective, *SHMT2*-mediated antioxidant defense may contribute to tumor adaptation against ferroptotic stress. In recent years, GPX4 has emerged as an important therapeutic target for ferroptosis-targeted cancer therapy [18]. Directly targeting GPX4 using novel small-molecule inhibitors, such as 2-oxopiperazine derivatives, has been demonstrated to potently induce ferroptosis and suppress tumor growth in oral cancer models [34]. While direct GPX4 inhibition blocks downstream lipid peroxide detoxification, our study highlights an alternative metabolic intervention strategy involving the upstream SHMT2–GSH axis. Combining direct GPX4 inhibition with SHMT2-targeted metabolic suppression may represent a potential therapeutic strategy for head and neck cancer.

The TCGA-HNSC analyses in this study are observational and cross-sectional. The co-expression of *SHMT2* with *GPX4*, *GCH1*, *DHODH*, and *FSP1* does not establish regulatory direction or a causal relationship. After multivariable adjustment, the association with advanced clinical stage was not statistically significant, whereas the association with clinical node positivity remained nominally significant. These findings should therefore be interpreted as exploratory clinical and transcriptional associations rather than mechanistic evidence.

Despite these findings, several limitations of the present study should be acknowledged. While our pharmacological and genetic rescue experiments support a contribution of ferroptosis to cytotoxicity following *SHMT2* loss, the partial protection conferred by apoptosis and necroptosis inhibitors indicates that SHMT2 suppression engages multifaceted cell-death pathways rather than ferroptosis exclusively. In addition, although SHMT2 suppression markedly decreased the GSH/GSSG ratio, this observation establishes disrupted glutathione redox balance rather than definitively proving that impaired SHMT2-derived glycine supply is the sole bottleneck for GSH biosynthesis. First, although erastin served as a robust mechanistic ferroptosis inducer, its unfavorable pharmacokinetic properties and potential systemic toxicity limit direct clinical applicability; therefore, erastin was utilized primarily as a functional probe rather than a therapeutic candidate. Second, although *GPX4* overexpression partially restored cell viability and reduced lipid peroxidation in *SHMT2*-knockdown cells, GPX4 rescue under erastin treatment could not be fully evaluated in the present study. The combined conditions of *SHMT2* knockdown, *GPX4* overexpression, and erastin treatment required further optimization of drug concentration and treatment duration, and sufficiently reliable results could not be obtained within the revision period. Therefore, further studies are required to determine whether GPX4 restoration can partially reverse the enhanced sensitivity to erastin caused by SHMT2 suppression. Third, intracellular iron status was further evaluated by quantifying Fe^2+^ levels (Figure S2E). Intracellular Fe^2+^ levels remained comparable between control and *SHMT2*-knockdown cells, suggesting that SHMT2 depletion did not markedly alter the measured intracellular Fe^2+^ content under the conditions examined. However, measurement of intracellular Fe^2+^ content alone does not directly establish the iron dependency of cell death. Further studies using complementary approaches will therefore be required to more directly establish the iron dependency of SHMT2 suppression-induced cell death. Fourth, the current study primarily utilized hypopharyngeal and oral cancer–derived HNSCC cell lines. Given the marked biological heterogeneity among head and neck cancer subtypes, including laryngeal and nasopharyngeal cancer, broader validation across HNSCC subtypes will be necessary. Fifth, the in vivo validation was limited by the small sample size (*n* = 4 per group), the absence of formal blinding, and the lack of preservation of the specific randomization sequence; nevertheless, animal procedures, daily monitoring, and tumor volume measurements were conducted jointly and cross-verified by co-authors to improve procedural consistency. Further validation in larger animal cohorts and complementary immunocompetent models would help establish the generalizability of the in vivo findings.

Importantly, the regulatory landscape of ferroptosis may become more complex under acquired therapeutic resistance. In the present study, SHMT2 suppression promoted ferroptosis primarily by disrupting the GSH–GPX4 antioxidant system, as reflected by a reduced GSH/GSSG ratio, decreased GPX4 expression, and increased lipid peroxidation. Nevertheless, the corresponding changes in GPX4 expression following SHMT2 suppression or overexpression, together with the partial rather than complete rescue achieved by GSH supplementation, raise the possibility that SHMT2 may influence GPX4-dependent antioxidant defense through additional regulatory mechanisms beyond its canonical role in serine–glycine metabolism. Notably, in a parallel study conducted by our group, ferroptosis-associated alterations were more pronounced in acquired chemoradiotherapy-resistant HNSCC models. These observations suggest that prolonged therapeutic pressure may reshape the ferroptosis-defense network and amplify the contribution of SHMT2-associated redox programs to tumor cell adaptation. Further investigation integrating treatment-sensitive models and models with acquired resistance will help define how this adaptive remodeling influences ferroptosis susceptibility and the response to ferroptosis-based combination strategies.

## 5. Conclusions

In summary, our study establishes mitochondrial *SHMT2* as a pivotal metabolic determinant of ferroptosis sensitivity in HNSCC. By maintaining the GSH/GSSG redox ratio and sustaining GPX4-mediated lipid peroxide detoxification, *SHMT2* protects cancer cells against lipid peroxidative damage. Pharmacological or genetic inhibition of *SHMT2*, particularly in combination with erastin, effectively overrides ferroptosis defense mechanisms and suppresses tumor progression. These insights define the SHMT2–GSH–GPX4 cascade as a critical therapeutic vulnerability, supporting *SHMT2*-targeted metabolic interventions to potentiate ferroptosis-inducing regimens in head and neck oncology.

## 6. Patents

A patent application resulting from the work reported in this manuscript has been filed and is currently pending.

## Data Availability

The TCGA-HNSC dataset analyzed in this study is publicly available from The Cancer Genome Atlas database via the Genomic Data Commons portal. Other data supporting the findings of this study are available from the corresponding authors upon reasonable request.

## Supplementary Materials

The following supporting information can be downloaded at: https://www.mdpi.com/article/10.3390/antiox15101300/s1, Figure S1: Effects of *SHMT2* suppression and sodium formate supplementation on HNSCC cell growth and viability; Figure S2: Effects of liproxstatin-1 and deferoxamine on cell death and viability in *SHMT2*-deficient HNSCC cells; Figure S3: Effects of *SHMT2* knockdown and SHIN2 treatment on GPX4 and xCT/SLC7A11 protein levels and *GPX4* mRNA expression in HNSCC cells; Figure S4: Effects of erastin and RSL3 on HNSCC cell viability and lipid peroxidation; Table S1: Primer sequences used for quantitative real-time PCR; Table S2: Target sequences used for SHMT2 knockdown; Table S3: Multivariable logistic regression analysis of advanced clinical stage; Table S4: Multivariable logistic regression analysis of clinical node positivity.

## Abbreviations

The following abbreviations are used in this manuscript:

HNSCC	Head and neck squamous cell carcinoma
SHMT2	Serine hydroxymethyltransferase 2
GPX4	Glutathione peroxidase 4
GCH1	GTP cyclohydrolase 1
DHODH	dihydroorotate dehydrogenase
FSP1	ferroptosis suppressor protein 1
GSH	Glutathione
ROS	Reactive oxygen species
qPCR	Quantitative real-time PCR
WB	Western blot
ANOVA	Analysis of variance
