# Supplementary material for: SHMT2 Suppression Disrupts Redox Homeostasis and Induces Ferroptosis Through the GSH–GPX4 Axis in Head and Neck Cancer
Source: Antioxidants (Basel). 2026 Oct 8;15(10):1300. doi: 10.3390/antiox15101300 (PMC13700825; doi:10.3390/antiox15101300)
Supplement: Supplementary file 1 [file antioxidants-15-01300-s001.zip › Supplementary_Figure_Legends.pdf]

Supplementary Figure Legends

Figure S1. Effects of SHMT2 suppression and sodium formate supplementation on HNSCC cell growth and viability

(A) Growth curves of SNU1041 and MSK-QLL1 cells transduced with control shRNA (shNC) or two independent shRNAs targeting SHMT2 (shSHMT2#1 and shSHMT2#2). Cell numbers were measured at 24, 48, and 72 h.

(B) SNU1041 and MSK-QLL1 cells were treated with DMSO, SHIN2 (80 μM), or SHIN2 (80 μM) plus sodium formate (1 mM) for 24 h. Cell viability was assessed by CCK-8 assay. Treatment groups are identified in the color legends and beneath the bars.

Data are presented as the mean ± SD from three independent biological experiments ($n = 3$). Data were analyzed using ordinary two-way ANOVA followed by Šídák's multiple comparisons test (A) or ordinary one-way ANOVA followed by Šídák's multiple comparisons test (B). ns, not significant; $*P < 0.05$, $**P < 0.01$, and $***P < 0.001$.

Figure S2. Effects of liproxstatin-1 and deferoxamine on cell death and viability in SHMT2-deficient HNSCC cells

(A) PI staining and flow cytometry of shNC and shSHMT2 SNU1041 and MSK-QLL1 cells treated with or without liproxstatin-1 (Lip-1, 200 nM). Relative PI-positive cells are quantified on the left; representative histograms are shown on the right.

(B) Cell viability of shNC and shSHMT2 cells treated with or without Lip-1 (200 nM), assessed by CCK-8 assay.

(C) PI staining and flow cytometry of shNC and shSHMT2 cells treated with or without deferoxamine (DFO, 100 μM). Relative PI-positive cells are quantified on the left; representative histograms are shown on the right.

(D) Cell viability of shNC and shSHMT2 cells treated with or without DFO (100 μM) for 72 h, assessed by CCK-8 assay.

(E) Relative intracellular Fe^{2+} levels following SHMT2 knockdown, measured using an iron assay kit.

Data are presented as the mean ± SD from three independent biological experiments (n = 3). Data were analyzed using ordinary one-way ANOVA followed by Šídák's multiple comparisons test. ns, not significant; *P < 0.05, **P < 0.01, and ***P < 0.001.

Figure S3. Effects of SHMT2 knockdown and SHIN2 treatment on GPX4 and xCT/SLC7A11 protein levels and GPX4 mRNA expression in HNSCC cells

(A) Protein expression of SHMT2, GPX4, and xCT/SLC7A11 in SNU1041 and MSK-QLL1 cells following genetic suppression of SHMT2 using shSHMT2 constructs, as determined by immunoblotting.

(B) Protein expression of GPX4 and xCT/SLC7A11 in SNU1041 and MSK-QLL1 cells after treatment with the dual SHMT1/2 inhibitor SHIN2 (40 or 80 μM) for 24 h, as determined by immunoblotting. *β*-Actin was used as a loading control. The numbers below the bands indicate relative band intensities quantified using ImageJ and normalized to β-actin, with the control group set to 1.00. Representative immunoblots from three independent biological experiments are shown.

(C，D) GPX4 mRNA expression measured by qRT–PCR after (C) SHMT2 knockdown or (D) SHIN2 treatment (40 or 80 μM, 24 h).

Data are presented as the mean ± SD from three independent biological experiments (*n* = 3). Data were analyzed using ordinary one-way ANOVA followed by Šídák's multiple comparisons test. ns, not significant.

Figure S4. Effects of erastin and RSL3 on HNSCC cell viability and lipid peroxidation

(A) Cell viability was assessed in SNU1041 and MSK-QLL1 cells treated with increasing concentrations of erastin (1–4 μM) for 24 h. Because 2 μM erastin had only a modest effect on cell viability in both cell lines, this concentration was selected for subsequent ferroptosis-sensitivity experiments.

(B) Cell viability of shNC and shSHMT2 cells treated with or without RSL3 (200 nM) for 24 h, assessed by CCK-8 assay.

(C) C11-BODIPY 581/591 flow cytometry of shNC and shSHMT2 cells treated with or without RSL3 (200 nM) for 24 h. Relative lipid peroxidation is quantified on the left; representative histograms are shown on the right.

Data are presented as the mean ± SD from three independent biological experiments ($n = 3$). Data were analyzed using ordinary one-way ANOVA followed by Dunnett's multiple comparisons test (A) or ordinary one-way ANOVA followed by Šídák's multiple comparisons test (B and C). ns, not significant; *$P < 0.05$, **$P < 0.01$, and ***$P < 0.001$.
