# Supplementary material for: SHMT2 Suppression Disrupts Redox Homeostasis and Induces Ferroptosis Through the GSH–GPX4 Axis in Head and Neck Cancer
Source: Antioxidants (Basel). 2026 Oct 8;15(10):1300. doi: 10.3390/antiox15101300 (PMC13700825; doi:10.3390/antiox15101300)
Supplement: Supplementary file 1 [file antioxidants-15-01300-s001.zip › Supplementary_Table.pdf]

Supplementary Tables

Table S1. Primer sequences used for quantitative real-time PCR

Gene name	Primer	Sequence (5′–3′)
SHMT2	Forward	TGGAGTAAATTGAGCTGCTG
SHMT2	Reverse	CTGGACATTGACTCCCCACT
PTGS2	Forward	AACACCCTCTATCACTGGCAT
PTGS2	Reverse	CAGCAATTTGCCTGGTGAATG
GPX4	Forward	GAGGCAAGACCGAAGTAAACTAC
GPX4	Reverse	CCGAACTGGTTACACGGGAA
GAPDH	Forward	ACCCAGAAGACTGTGGATGG
GAPDH	Reverse	TTCTAGACGCAGGTCAGGT

Note: SHMT2, serine hydroxymethyltransferase 2; PTGS2, prostaglandin-endoperoxide synthase 2; GPX4, glutathione peroxidase 4; GAPDH, glyceraldehyde-3-phosphate dehydrogenase. Primer sequences are shown in the 5′ to 3′ direction.

Table S2. Target sequences used for SHMT2 knockdown

Target	Sequence (5′–3′)
shSHMT2#1	AAGACTGCCAAGCTCCAGGATTTCAAATC
shSHMT2#2	AAGTCAAAGCACACCTGCTGGCAGACATG

Note: Target sequences are shown in the 5′ to 3′ direction.

Table S3. Logistic regression models of SHMT2 expression and clinical features in TCGA-HNSC

Advanced clinical stage (III–IV vs I–II)

Complete cases: n = 469; 364 advanced-stage and 105 early-stage cases.

**Variable and comparison**	**Model 1**<br>**Unadjusted**	**Model 2**<br>**Clinical adjustment**	**Model 3**<br>**Full adjustment**
SHMT2 expression<br>(per 1-unit log2[RSEM + 1] increase)	1.82 (1.23–2.69)<br>P = 0.003	1.57 (1.03–2.39)<br>P = 0.034	1.49 (0.98–2.29)<br>P = 0.065
Age (per 1-year increase)	—	0.99 (0.97–1.01)<br>P = 0.357	0.99 (0.97–1.01)<br>P = 0.395
Sex: male vs female	—	1.44 (0.87–2.37)<br>P = 0.155	1.39 (0.84–2.29)<br>P = 0.201
HPV status: positive vs negative	—	1.58 (0.67–3.75)<br>P = 0.299	1.34 (0.55–3.27)<br>P = 0.522
Site: oral cavity vs.<br>larynx/hypopharynx	—	0.41 (0.21–0.78)<br>P = 0.007	0.42 (0.22–0.80)<br>P = 0.009
Site: oropharynx vs.<br>larynx/hypopharynx	—	0.33 (0.13–0.87)<br>P = 0.026	0.35 (0.13–0.93)<br>P = 0.035
Tumor purity<br>(per 0.10 absolute increase)	—	—	1.14 (0.99–1.30)<br>P = 0.068
Clinical T category:<br>T3–T4 vs T1–T2	—	—	—

Table S3 legend.

Values are odds ratios (95% confidence intervals), followed by two-sided Wald P values. —, not included in the model. Intercepts are omitted.

Model 1 includes SHMT2 expression only and does not present separate univariable models for each covariate. Model 2 adds age, sex, HPV status and anatomical site; Model 3 additionally adds ABSOLUTE tumor purity. T, N and M categories were not included in the overall-stage models.

SHMT2 ORs are per 1-unit increase in log2(RSEM + 1), approximately a doubling of expression at the observed levels. Age is per year. Tumor purity is measured on a 0–1 scale, with ORs reported per 0.10 absolute increase (10 percentage points). Rescaling the original standardized SHMT2 and purity coefficients does not change P values or model fit.

Reference categories are female sex, HPV-negative status and larynx/hypopharynx. Sites were grouped as oral cavity, oropharynx and larynx/hypopharynx because no hypopharyngeal early-stage cases were available. The same complete cases were used across models; unknown stage and missing covariates were excluded.

Table S4. Logistic regression models of SHMT2 expression and clinical features in TCGA-HNSC

Clinical lymph-node positivity (N1–N3 vs N0)

Complete cases: n = 460; 232 node-positive and 228 node-negative cases.

**Variable and comparison**	**Model 1<br>Unadjusted**	**Model 2<br>Clinical adjustment**	**Model 3<br>Full adjustment**
SHMT2 expression<br>(per 1-unit log2[RSEM + 1] increase)	1.62 (1.18–2.22)<br>P = 0.003	1.62 (1.16–2.28)<br>P = 0.005	1.55 (1.09–2.21)<br>P = 0.014
Age (per 1-year increase)	—	0.97 (0.95–0.99)<br>P <0.001	0.97 (0.95–0.99)<br>P <0.001
Sex: male vs female	—	0.99 (0.63–1.56)<br>P = 0.980	0.93 (0.58–1.49)<br>P = 0.767
HPV status: positive vs negative	—	1.47 (0.72–2.98)<br>P = 0.291	1.57 (0.75–3.31)<br>P = 0.235
Site: oral cavity vs<br>larynx/hypopharynx	—	0.98 (0.61–1.56)<br>P = 0.929	1.19 (0.73–1.93)<br>P = 0.491
Site: oropharynx vs<br>larynx/hypopharynx	—	1.58 (0.72–3.47)<br>P = 0.256	2.46 (1.06–5.69)<br>P = 0.036
Tumor purity<br>(per 0.10 absolute increase)	—	—	1.03 (0.91–1.16)<br>P = 0.673
Clinical T category:<br>T3–T4 vs T1–T2	—	—	2.84 (1.83–4.43)<br>P <0.001

Table S4 legend

Values are ORs (95% CIs) with nominal two-sided Wald P values; —, not included in the model. Intercepts are omitted. Model 1 includes SHMT2 expression only; Model 2 adds age, sex, HPV status and anatomical site; Model 3 additionally adds ABSOLUTE tumor purity and clinical T category (T3–T4 vs T1–T2). Overall stage was excluded because it incorporates nodal information. Other units and reference categories are as in Table S3.

The reanalysis began with 515 primary tumors with expression data. HPV status was based on HNSC_HPV+ and HNSC_HPV− annotations rather than a uniform clinical assay.

P values were not adjusted for multiple testing. Unknown N status (NX) and missing covariates were excluded. Covariate estimates are conditional associations, not standalone causal effects. Clinical N status does not imply pathological confirmation; staging uses historical AJCC annotations. This public-data reanalysis differs from the original manuscript analysis of 520 tumors with TPM expression.

Data sources: cBioPortal study hnsc_tcga_pan_can_atlas_2018 (www.cbioportal.org); TCGA.HNSC.sampleMap/HNSC_clinicalMatrix (tcga.xenahubs.net); GDC PanCancer Atlas ABSOLUTE purity file (gdc.cancer.gov/about-data/publications/pancanatlas). Accessed 17 September 2026.
